# Fermentation-based oxalic acid production aimed at sustainable critical metal recovery from electronic waste

**DOI:** 10.1007/s00253-026-14040-4

**Published:** 2026-09-23

**Authors:** Aylin Nur Erkmen, Roland Ulber, Thomas Jüstel, Mirjam Altendorfner

**Affiliations:** 1 Department of Chemical Engineering, FH Münster University of Applied Sciences, Stegerwaldstrasse 39, 48565 Steinfurt, Germany; 2 Department of Bioprocess Engineering, RPTU University of Kaiserslautern-Landau, Gottlieb Daimler Strasse 49, 67663 Kaiserslautern, Germany

**Keywords:** Oxalic acid, ATCC1015, Submerged fermentation, Fed-batch, Particle, Morphology, Bioleaching

## Abstract

**Abstract:**

This study systematically investigates the biogenic production of oxalic acid (OA, H_2_C_2_O_4_) via submerged fermentation to establish a sustainable reagent for critical metal recovery applications. Preliminary shake-flask experiments evaluated *Aspergillus niger* strains (ATCC 1015 and CECT 2807), optimal carbon sources, and the influence of pH adjustment. Despite inherent biological variance across replicates, *A. niger* ATCC 1015 demonstrated a superior production trajectory, yielding 71.0 ± 27.7 mM OA in 5 days. Intermittent pH adjustment above 4.0 significantly enhanced secretion to 90.4 ± 5.8 mM, while glucose was identified as the optimal carbon source (Y_P/S_ ≈ 0.4 g/g). Building upon these preliminary findings, process intensification was conducted in a 10 L stirred-tank bioreactor. Transitioning to a fed-batch strategy with pulsed feeding and continuous pH control effectively contributed to substantially higher OA titers, achieving a peak OA titer of 260.1 ± 4.8 mM over 14 days. Co-production of gluconic acid and acidogenesis inhibition posed a challenge in attaining higher product yields. Collectively, these findings establish a robust, scalable bioprocess for sustainable OA generation, directly supporting its emerging application as a highly selective leaching agent for critical metal recovery from electronic waste streams.

**Key points:**

- *A.niger strain ATCC1015 was pinpointed as a suitable strain for biogenic OA production*
- *Fed-batch with pulsed feeding maximizes OA titer in scale-up.*
- *Gluconic acid and phosphate excess hinder selective OA production.*

**Supplementary Information:**

The online version contains supplementary material available at https://doi.org/10.1007/s00253-026-14040-4.

## Introduction

Oxalic acid (sum formula: H_2_C_2_O_4_) is a dicarboxylic organic acid widely used in various industrial branches, among which the pharmaceutical industry, agriculture, textile and leather, and chemical and food industries can be counted (Schuler et al. 2021). The first synthesis of OA was established by Scheele in 1776 through sugar oxidization utilizing nitric acid, followed by an alternative protocol developed by Wöhler using cyanogen hydrolysis in 1824 (Verma et al. 2019). At present, OA is predominantly produced through chemical synthesis routes such as propylene, ethylene oxidation, and dialkyl oxalate processes, while Rhône-Poulenc is the largest manufacturer on a global scale (Schuler et al. 2021).

**Table 1:** Summary of literature studies on OA production parameters and operational strategies

Strain ID	Vessel	C-source	Mode	Adopted strategy	OA [mM]	Reference
WU-2223	Shake-flask	Glucose	Batch	pH > 4.0	321	Kobayashi et al. (2014)
B60	Shake-flask	Glucose	Batch	pH > 4.0	88.9	Kubicek et al. (1988)
ATCC 6275	Shake-flask	Whey	Batch	C (%)^a^	4.0	Brown et al. (2018)
ATCC 9029	Shake-flask	Whey	Batch	C (%)^a^	7.0	Brown et al. (2018)
CMB 120.49	STR (2 L)	Sucrose	Fed-batch	pH > 4	427.0	Strasser et al. (1994)
1120	Shake-flask	Whey	Batch	pH ≈ 6.0	410.9	Santoro et al. (1999)
1120	Biostat E (10 L)	Whey	Batch	pH ≈ 6.0 kLa^b^	459.9	Bohlmann et al. (1998)
1120	Biostat E (10 L)	Whey	Batch	pH ≈ 6	375.4	Cameselle et al. (1998)
1120	Biostat E	Whey	Batch	pH ≈ 6.0	375.4	Cameselle et al. (1998)
XP^c^	STR (2 L)	Rapeseed oil	Batch	pH ≈ 5	764.2	Rymowicz and Lenart (2003)
W78C	Biostat A (6 L)	Sucrose	Batch	pH > 6	714.2	Walaszczyk et al. (2018)
F22	BioCnS (50 L)	Fructose	Batch	kLa	83.1	Lee et al. (2018)
NJDL-12	Shake-flask	Glucose	Batch	pH = 6.5	26.1	Li et al. (2016)
NJDL-03^d^	Shake-flask	Glucose	Batch	pH = 6.5	10.0	Li et al. (2016)

^a^ C(%): Adjustment in the concentration of the carbon source

^b^$k_La$: Optimization through the kLa value by adjusting the stirrer speed (rpm) and aeration (vvm)

^c^ XP: Modified *A.niger* strain through UV-mutagenesis

^d^NJDL-03: *Penicillium oxalicum* strain

Filamentous fungi, specifically *Aspergillus sp.* and *Penicillium sp.*, are widely used as microbial cell factories for the secretion of organic acids such as citric, oxalic, and gluconic acid through their metabolic processes (Gadd and Gadd 1999). Notably, owing to its high productivity and industrial viability, *Aspergillus niger* is extensively used for citric acid fermentation (Książek 2023). Early observations by Wehmer (1893) laid the foundation by distinguishing *Aspergillus niger* as an OA accumulator compared to *Penicillium glaucum*, which was later broadened by Currie and Thom (1915) to include *Penicillium oxalicum* (Currie and Thom 1915).

Further research into the metabolism of *Aspergillus niger* revealed a cytoplasmic, TCA-independent pathway for oxalate formation mediated by the enzyme *oxaloacetate hydrolase (OAH)* (Kubicek et al. 1988). Kubicek et al. (1988) pinpointed the C-C splitting of oxaloacetate through *OAH* enzyme at pH levels above 4 (Kubicek et al. 1988). In the same vein, Ruijter et al. (1999) demonstrated citric acid production by *A.niger* mutant lacking *OAH* at pH 5 in the presence of manganese ions, conditions that are typically inhibitory to the wild type (Ruijter et al. 1999). In light of these findings regarding the cytoplasmic pathway driven by *OAH* enzyme, several studies have tackled the optimization of OA secretion by *A. niger* at different scales, adopting various methodologies with a primary focus on carbon source and pH regulation (Strasser et al. 1994; Cameselle et al. 1998). The key findings from these fermentation studies are outlined in Table 1.

While the OA secretion by *Aspergillus niger* is well-documented, the primary industrial application of *A. niger* has been for citric acid fermentation, where research efforts are often directed toward the suppression of OA as a by-product to maximize target yields (Gadd and Gadd 1999; Książek 2023). Recently, metal recycling represents another novel application area for OA, garnering significant attention in light of escalating electronic waste (e-waste) crisis and growing concerns regarding the long-term supply of critical metals, such as gallium (Ga) and rare earth elements (REEs), which are indispensable for manufacturing electronic devices such as light-emitting diodes (LEDs), tablets, smartphones, and so on. Therefore, OA serves as a strategic and sustainable alternative to conventional inorganic reagents, which are used in on-going metal recycling operations, such as hydrochloric acid (HCl) and sulfuric acid ($H_{2}SO_{4}$), for dissolving end-of-life (EoL) devices and recovering these valuable materials.

Specifically, OA establishes robust octahedral coordination compounds with hard metals via complexolysis, specifically trivalent metals such as Al(III), Fe(III), Ga(III), and so on, and serves as a selective precipitant for divalent metal ions such as for REE (Weber and Birgit 2023; Verma et al. 2019). Erkmen et al. (2025b) pinpointed that OA under optimized conditions (710.55 acid concentration, at 84.5 $^\circ$C, 50 g/L of waste loading) could recover up to 37% Ga from waste minerals (Erkmen et al. 2025a). This supports the earlier findings by Zhou et al. (2019) who demonstrated a 90.36% of Ga recovery from waste surface-mounted light-emitting diodes (SMD-LEDs) under optimized conditions at 90 $^\circ$C, 10 g/L solid loading, and 700 mM acid concentration (Zhou et al. 2019). In this context, OA presents a promising solution owing to its dual functionality, enabling a dissolution and robust formation of complexes, which is alluring for targeted metal recovery (Verma et al. 2019; Erkmen et al. 2025a; Gerold et al. 2024).

Nonetheless, despite offering a promising alternative compared to conventional harsh reagents, Choi et al. (2022) pinpointed that OA failed to meet the classification criteria of green chemicals (Choi et al. 2022). Their assessment considered both a metal recovery (%) and toxicity (LD_50_), revealing OA exhibiting a higher LD_50_ than sulfuric acid (H_2_SO_4_), raising concerns about its environmental impact (Choi et al. 2022). This conundrum is rooted in the entire life-cycle of OA, as it is primarily derived from petrochemical processes, which are associated with volatile intermediates and significant CO_2_ emissions (Schuler et al. 2021).

Aligning with the aims of the European Union (EU) to achieve climate neutrality by year 2050 by prioritizing renewable energies and transitioning from fossil feed-stocks into the sustainable alternatives for material production gives an incentive for biogenic oxalic acid production as well as sustainable metal recovery from waste resources (Commission 2019; Schuler et al. 2021). In this regard, fermentation processes involving filamentous fungi emerge as a viable and sustainable strategy to address both 350,000-ton annual OA demand and the crisis in critical raw material supply, promoting selective secondary production based on a “key-lock” principle (Schuler et al. 2021; Erkmen et al. 2025a).

To bridge this gap, this research addresses the aforementioned limitations by establishing a comprehensive framework for submerged OA fermentation by *Aspergillus niger*. Through a systematic study including strain comparison, pH regulation, process intensification on a larger scale (10 L) whilst evaluating different fermentation modes, this study establishes fundamentals for an industrially viable OA production. The developed process is designed to yield a biogenic agent rich in OA, specifically conceptualized for the recovery of critical metals such as gallium from e-waste and other secondary waste streams, leveraging the enhanced performance often attributed to synergistic secondary metabolites in fungal fluids (Maneesuwannarat et al. 2016). Consequently, by transitioning away from petrochemical routes, this study positions fungal fermentation as a stand-alone green technology, contributing to sustainable bioprocess development for strategic metal recovery in alignment with the European Union’s 2050 circular economy goals.

## Materials and methods

### Strain, subculture, and inoculum preparation

*Aspergillus niger* strains used in this study included ATCC 1015 (LGC Standards) and CECT 2807 (University of Valencia culture repository). Accordingly, strains were subcultured periodically (5–7 days) on potato-dextrose agar (PDA) plates under sterile conditions and incubated at 30 $^\circ$C. PDA composition: 4 g/L Potato infusion; 20 g/L Glucose; 15 g/L Agar; pH 5.6 ± 0.2 (from *Carl Roth GmbH+Co. KG*; acc. to Ph. Eur./USP, for microbiology)

For spore inoculum preparation, cultures were aseptically scraped from PDA plates using sterile disposable scalpels into a solution of 0.9 N KCl and 10 μL/L Triton-X. Spore concentrations were determined using a Neubauer-improved hemocytometer via serial dilution, in which inoculum concentrations were adjusted to 10^6^-10^7^ spores/ml for shake-flask experiments and 10^8^ spores/mL for fermenter experiments. Pre-cultures in glycerol solutions were stored in cryo-vials at -20 $^\circ$C.

### Fermentation experiments

#### Shake-flask experiments

The nutrient media used in the shake-flask experiments were adapted from Bosshard et al. (1996) and contained (g/L): Glucose (30), NaNO_3_ (1.5), KH_2_PO_4_ (0.5), MgSO_4_.7H_2_O (0.025), KCl (0.025), yeast extract (1.6) (Bosshard et al. 1996). This medium was supplemented with 100 mM phosphate buffering, and the pH was adjusted to 6.0 ± 0.2 with concentrated (3 N) NaOH. All reagents were of analytical grade. Sterilization of nutrient and agar media was performed in closed sterile vessels at 120 $^\circ$C for 20 min under 1.5 psi pressure. Preceding the inoculum, 99 ml of sterile nutrient media was aseptically dispensed into 250 ml baffled *Duran* Erlenmeyer flasks under a vertical laminar flow hood. Subsequently, each flask was inoculated with spore solution (10^6^ spores/mL) to a final inoculum density of 1% (v/v).

Sampling was conducted aseptically on sporadic intervals spanning over 240 h using sterile pipette tips and sample holders to prevent cross-contamination. pH fluctuations and OA concentration were measured throughout the experiment. In buffering tests, pH was maintained at 6.0 ± 0.2 using 3 N NaOH. At each interval, the $\text {pH}$ of the culture medium was monitored using a digital $\text {pH}$ meter with a DO sensor, calibrated prior to use with $\text {pH}$ 4.0 and 7.0 standard buffers.

All shake-flask experiments were performed in biological triplicate (*n* = 3). Error bars represent the standard deviation of the mean. To account for the loss in nutrient solution volume as a result of intermittent sampling and evaporation during cultivation, upon sampling, double-distilled water (ddH2O) was added under aseptic conditions to maintain the total volume of 100 ml.

Following fermentation, fungal pellets were separated from the biogenic supernatant solutions using Nalgene vacuum filters (0.2 μm). The cell-free supernatant was stored at 4 $^{\circ }$C for subsequent glucose and organic acid analysis.

#### Fermenter (10 L) experiments

A Biostat-C (Sartorius AG, Göttingen, Germany) bioreactor with a 15-L total volume (10-L working volume) and an H:D ratio of 1:3 was used for all large-scale fermentation experiments. The nutrient medium, adapted from Bosshard et al. (1996), contained (g/L): Glucose (150), NaNO_3_ (1.5), KH_2_PO_4_ (0.5), MgSO_4_·7 H_2_O (0.025), KCl (0.025), and yeast extract (1.6) (Bosshard et al. 1996). This was further supplemented with 100 mM phosphate buffering, and the pH was maintained at 6 ± 0.2 using concentrated (3 N) NaOH. All reagents used were of analytical grade.

Mixing was provided by a triple six-blade Rushton turbine impeller, and aeration was supplied through a ring sparger. The reactor temperature was controlled via a cooling jacket. Process parameters including temperature, volumetric airflow (L/min), dissolved oxygen (DO (%)), and pH were continuously monitored and controlled using an integrated amplifier system. The DO level was maintained using a cascade control system that adjusted the stirrer speed and/or aeration rate.

The pH was measured and controlled using a Mettler-Toledo S7 pH sensor, pre-calibrated using analytical pH 4 and 7 buffer solutions. Dissolved oxygen was measured with a Hamilton OxyFerm (pO_2_) sensor, calibrated with a two-point method (0% after nitrogen sparging and 100% after full aeration).

Sterilization of the fermenter was performed in three stages (heating, sterilization, cooling) using the sterilization mode. The sparging was adjusted to sterilization mode, and the aeration valve and cooling water were turned off. Sterilization occurred at 120 $^{\circ }$C and up to 1.5 atm of gauge pressure for 25 min. During the cooling step, cooling water was turned on once the reactor temperature reached 85 $^{\circ }$C to expedite the process.

For fermentation experiments, the vessel was initially filled with 8.8 L of nutrient medium, accounting for ≈ 0.8 L loss during sterilization. After sterilization, 0.2 v/v % Pluronic F68 (foam-suppressing agent) and 0.1 N buffer solution were added through a syringe equipped with a 0.2 μm membrane filter. An inoculum of 1%, prepared from pre-grown pellet cultures (72–96 h exponential phase), was transferred aseptically from sterile culture flasks to the fermenter via a peristaltic pump. To enhance OA production, filter-sterilized (0.2 μm) 3 N NaOH was integrated via peristaltic pumps after the initial lag and growth phase (48 h) to maintain the fermentation medium pH at 6 ± 0.2. The following operational modes were investigated:

- **Batch:** The fermentation was performed with an initial glucose concentration of 150 g/L without further feeding.
- **Fed-batch:** Based on preliminary data, a pulsed feeding strategy was employed. After an initial batch phase (initial glucose concentration: 30 g/L), pulses of a concentrated glucose solution (stock concentration: 300 g/L) were added at 72-h intervals to maintain a target substrate level, with the total glucose added over the 15-day fermentation for constituting the total sum of 150 g/L.

Samples (20–25 mL) were withdrawn aseptically at 24-h intervals via a steam-sterilized port at the base of the bioreactor to monitor residual glucose and organic acid concentrations. Biomass was not quantified due to the dispersed mycelial morphology and the difficulty in accurately subtracting the pre-grown inoculum mass.

#### Glucose and organic acid determination

Collected samples were syringe-filtered (0.45μm) directly and analyzed for residual glucose using the dinitrosalicylic acid (DNS) assay (Miller 1959). The DNS reagent was prepared by dissolving 10 g of DNS and 300 g of Rochelle salt in 800 mL of 0.5 N NaOH, with the final volume complemented to 1 L with double-distilled H_2_O. For the DNS assay, 100 μL of the diluted sample was mixed with 400 μL of the prepared DNS reagent. The mixture was heated in a heating block for 5 min at 95 $^{\circ }$C. Subsequently, samples were promptly placed in an ice bath. Next, the prepared samples for glucose quantification were pipetted into a sterile F-type 96-well plate, in which the absorbance at 570 nm using a micro-plate reader was measured.

OA concentration in the filtrate was determined via Capel-105 M fully automated capillary electrophoresis instrument (Lumex Instruments, Canada). Direct samples from the fermentation liquid were syringe-filtered (0.45μm) and serially diluted with ddH_2_O. Before sample analysis, daily conditioning on Capel-105 M was performed, which comprised the serial flushing of capillaries with 1 N NaOH, ddH_2_O, and a buffer solution consisting of chromium (VI) oxide (10 mM), diethanolamine solution (30 mM), and Hexadecyltrimethylammonium hydroxide (CTA-OH) (3 mM). Subsequently, the samples were loaded into the autosampling unit and hydrodynamically injected into the capillary at 30 mbars for 5 s for analysis. Samples were then hydrodynamically injected (30 mbars for 5 s) and analyzed at -25 kV for 8–10 min, with indirect UV detection at 254 nm. Analyte concentrations were calculated via peak area against a linear regression of analytical standards (oxalic, citric, gluconic acids and nitrate, phosphate, chloride, sulfate ions). All measurements were performed in triplicate (95% CI).

While this comprehensive suite of analytes was monitored throughout all cultivations to ensure nutrient sufficiency and metabolic profiling, only components exhibiting significant quantitative accumulation or demonstrating a direct influence on the fermentation kinetics are reported. For the preliminary shake-flask experiments, secondary organic acids remained below the quantification limit and are therefore omitted.

## Results

### Comparative study of two strains for biogenic oxalic acid production

**Fig. 1:**
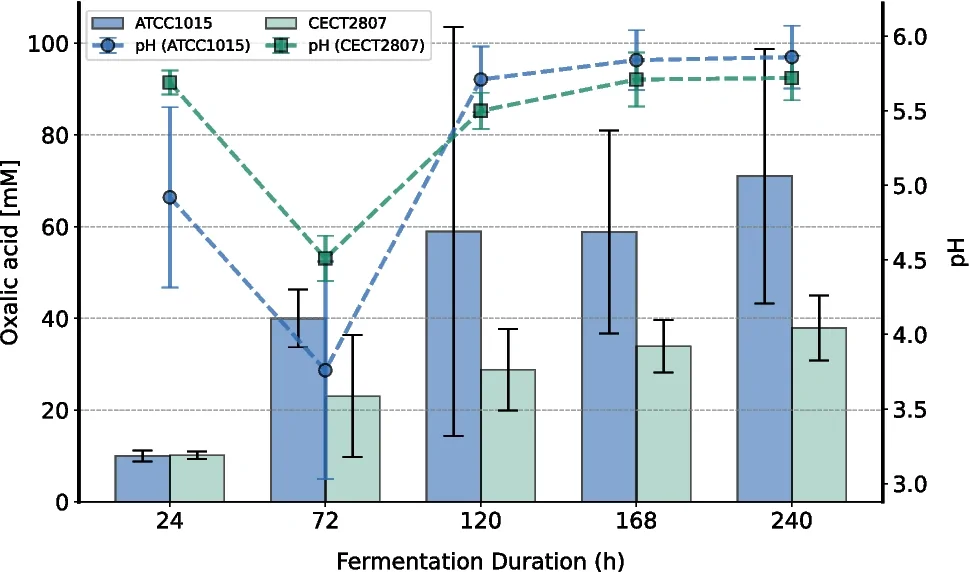
OA secretion and the detected pH during comparative strain test in small-scale shake fermentation experiments. In the bar chart stacked with a line plot, bars correspond to the secreted OA amount (mM), and lines to the measured pH in the secondary *y*-axis (pH_initial_ = 6.0 ± 0.2). The data represents the mean of biological replication experiments (*n* = 3). Error bars on both the bar and line plot denote the standard deviation of the biological triplicates

To differentiate the OA production capabilities between the selected *A. niger* fungal strains, small-scale batch fermentation experiments were conducted and monitored over a 240-h period. Figure 1 presents the comparative analysis of OA secretion (mM) and pH fluctuations between ATCC1015 and CECT2807 strains.

As outlined in Fig. 1, ATCC1015 showed a notably higher mean OA secretion compared to CECT2807 throughout the fermentation spanning over 240 h. While no clear distinction could have been made in terms of acid production in the initial phase (24 h), ATCC1015 exhibited rapid OA secretion in the latter sampling times, reaching a peak at 71.0 ± 27.7 at 240 h. The trend in acid peak was corroborated by the change in pH, in which ATCC1015 exhibited sharp decline in the pH (pH = 3.8 ± 0.7) at 72 h, whereas the strain CECT2807 exhibited a relatively lower pH decline at the same sampling time (pH = 4.5 ± 0.2). After sampling at 72 h, pH of the fermentation medium was adjusted to 6.0 ± 0.2 to prevent the suppression of *OAH* enzyme, leading to a progress in the OA secretion in the latter stages of the fermentation.

### pH regulation

As numerous studies attributed the significant biogenic OA secretion to the pH regulation by keeping pH > 4, complementary to the strain comparison, sporadic pH adjustment on days 1, 4, and 7 was implemented in the small-scale shake cultures. In addition, a separate fermentation batch was conducted where the culture medium’s pH was maintained at a neutral state (pH 6.0 ± 0.2) throughout the experiment. Owing to the prolonged lag phase in this set of experiments (≈ 26–28 h), neither acid secretion nor pellet formation could be detected at 24 h.

**Fig. 2:**
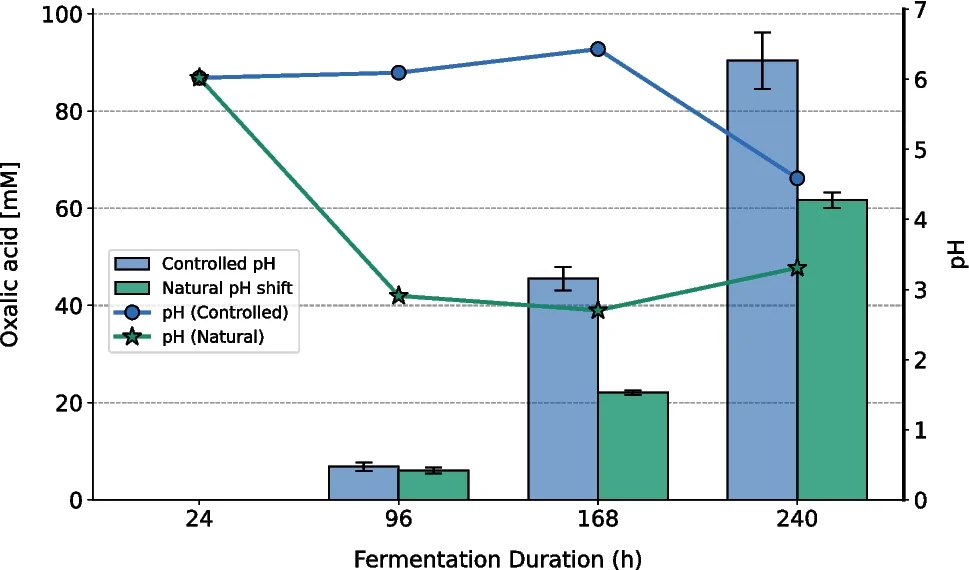
The influence of pH regulation on OA secretion in shake-flask cultures. The bars (primary *y*-axis) represent the mean concentration of secreted OA (mM) in cultures with intermittent pH control (“Controlled pH”) versus unregulated cultures (“Natural pH shift”). The lines (secondary *y*-axis) indicate the measured pH at each sampling point over the 240-h fermentation. All data points represent the mean of three biological replicates (*n* = 3), with error bars indicating the standard deviation

As shown in Fig. 2, following the OA production induction phase (≈ 24 h), both groups experienced a significant drop in pH to ≈ 2.9 and secreted comparable OA by 96 h. After adapting the initial pH regulation on day 4 (96 h), the pH-regulated cultures demonstrated a ≈ 7-fold surge in the secretion at 168 h, ultimately reaching a final titer of 90.4 ± 5.8 mM acid at 240 h.

On the other hand, the control group, where no pH regulation was integrated, secreted notably lower OA. As indicated on the secondary *y*-axis of Fig. 2, the pH of the non-regulated group remained below the critical threshold at pH 4, suppressing therefore the regulation of the *OAH* enzyme. As the fermentation progresses toward 168 h, the discrepancy in acid generation by both groups were doubled and persisted until the finalization of the fermentation at 240 h.

Thus, these results confirm that even sporadic pH regulation on small-scale batch cultures is a highly effective strategy for boosting OA yields.

**Fig. 3:**
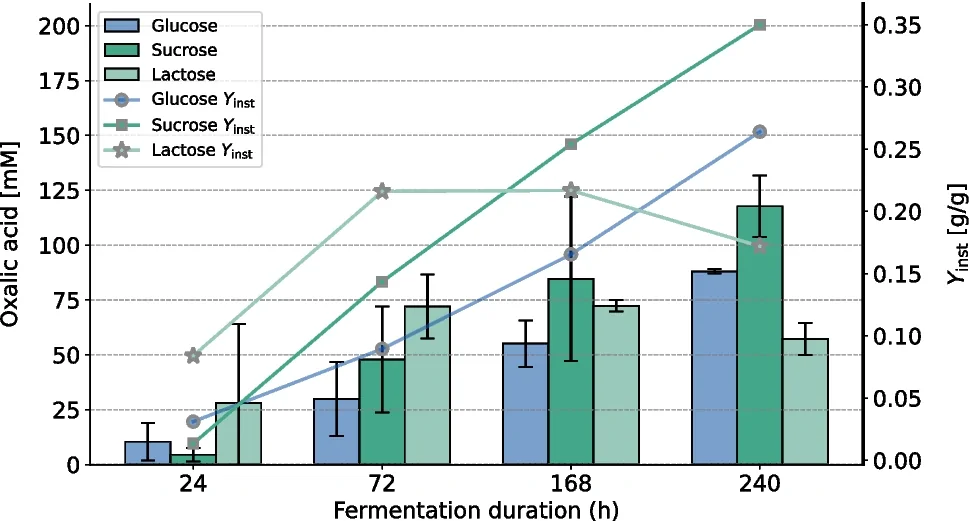
Comparison of carbon sources for OA secretion. The primary *y*-axis (bars) corresponds to the secreted OA concentration (mM), while the secondary *y*-axis (lines) represents the product yield on substrate (Y_P/S_, (g/g)). All data points represent the mean of three biological replicates (*n* = 3), with error bars indicating the standard deviation

**Fig. 4:**
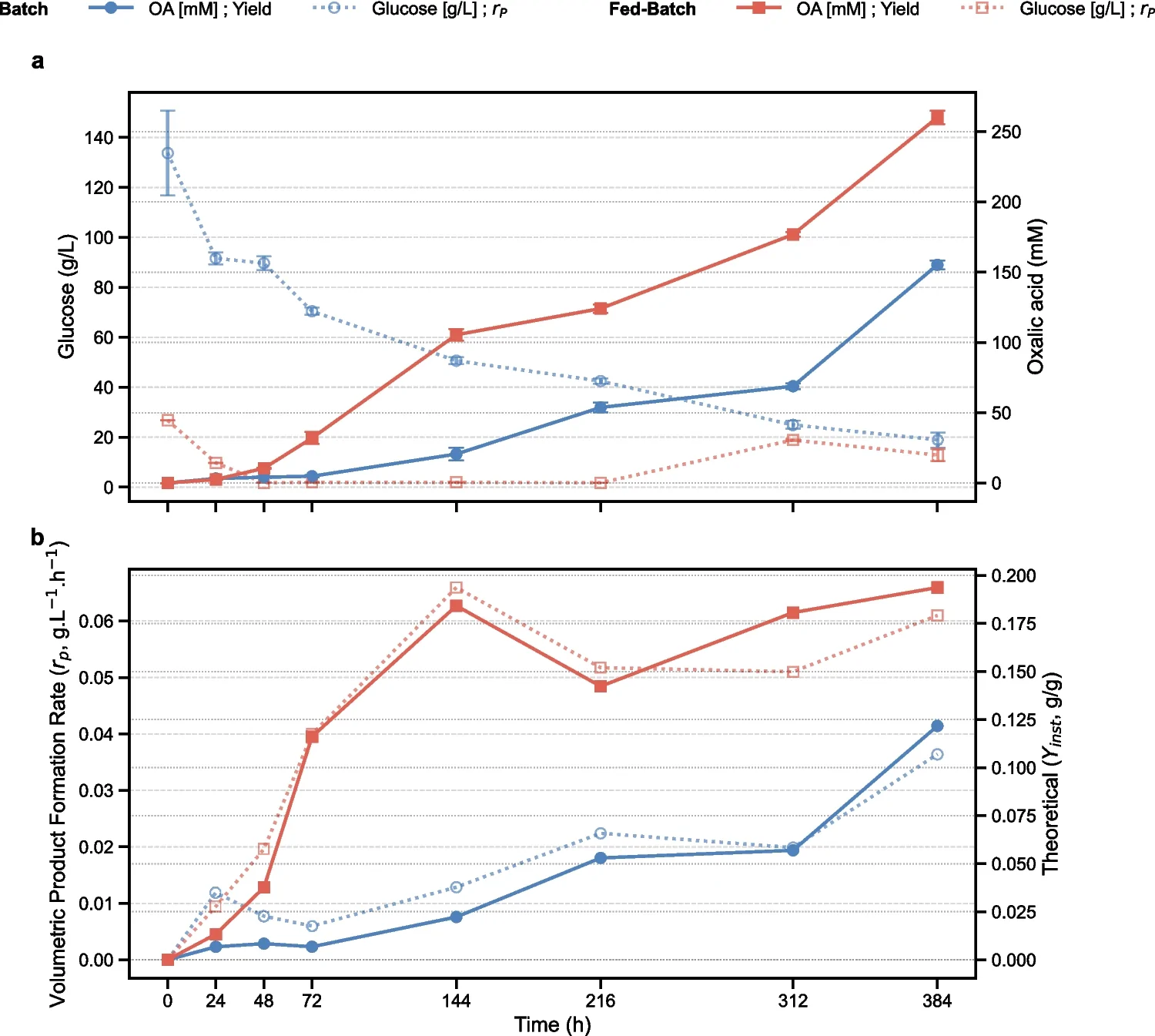
**Comparative performance of different fermentation strategies.** The panels illustrate key process parameters over time for batch, fed-batch, and repeated fed-batch operations. **a**) Glucose consumption profile. **b**) OA production profile. **c**) Instantaneous product yield ($Y_{\text {inst}}$). Data are presented as the mean ± standard deviation (n=3)

### Carbon source

To determine the impact of carbon source on the OA generation in the shake cultures of *A. niger*, the media described in the methods and materials were adjusted by implementing glucose, sucrose, and lactose at 30 g/L for each. As given in Fig. 3, the choice of substrate influenced the OA generation and the overall product yield per g substrate.

The choice of carbon source significantly influenced both the final OA titer and the product yield. As shown in Fig. 3, glucose proved to be the most effective substrate over the 240-h fermentation, resulting in the highest titer (136.9 ± 69.4 mM) and a product yield (Y_P/S_) of 0.4 (g/g). Fermentation on sucrose resulted in a final yield of 82.8 ± 55.0 mM (Y_P/S_=≈ 0.25), which was notably lower than that of glucose. In contrast, lactose was the least effective substrate, with production peaking at approximately 72.1 ± 14.6 mM (Y_P/S_=≈ 0.2) by 72 h before a subsequent decline.

These results in Fig. 3 suggest that while ATCC1015 can metabolize all three sugars, its metabolic pathways most efficiently channel the monosaccharide glucose towards OA synthesis over the full course of the fermentation. In contrast, the conversion of sucrose and lactose appeared to be less efficient or become inhibited as the fermentation progressed.

### Larger scale oxalic acid fermentation: process intensification across different operational modes

Building on the preliminary shake-flask experiments, process intensification experiments aimed at generating higher oxalate titers on a larger fermenter scale (10 L) were performed in batch and fed-batch operational modes. The performance of each strategy was assessed in terms of substrate consumption (g/L), the secreted OA amount (mM), product formation rate ($r_{\textit{P}}$), and attained theoretical yield (Y_P/S_). Figure 4 presents the 384-h fermentation profiles in both processes, allowing a direct comparison of the different fermentation strategies.

As shown in Fig. 4, OA fermentation in batch mode exhibited distinct phases over the 384-h process. Following an initial lag phase until ≈ 72 h, after which OA was gradually secreted. A transition to an accelerated production phase occurred only after 312 h, which coincided with the residual glucose concentration falling below ≈ 20 g/L. By the end of fermentation (384 h), the final OA titer reached 155.2 mM, corresponding to an overall volumetric product formation rate (r_P_) of 0.036 g.L^.1^.h^-1^.

The fed-batch strategy, utilizing intermittent pulsed feeding, effectively promoted acid secretion following the initial lag phase (≈ 28–30 h). This approach resulted in a cyclical production pattern comprising a gradual and consistent surge in the acid secretion, corresponding to 72 h intervals of pulse feeding. By the end of the second cycle at 144 h, the titer has already reached ≈ 100 mM (r_P_ ≈ 0.06 g.L^-1.^h^-1^).

In the final exponential production phase (312–384 h), glucose consumption plateaued, and r_P_, the volumetric production rate, signaled a stagnation, indicating the end of the productive period.

**Table 2:** Comparison of theoretical yields, volumetric product formation rates, and secreted oxalic acid (OA) titers across fermentation modes at 384 h

Mode	$Y_{P/S}$ (g/g)^a^	$r_P$ (g L$^{-1}$ h$^{-1}$)	OA (mM)
Batch	0.122	0.036	155.2 ± 3.1
Fed-batch	0.194	0.061	260.1 ± 4.8

^a^ Theoretical product yield representing the maximum conversion rate of substrate into OA, assuming all metabolic activity is channeled into product formation

Overall, the fed-batch strategy significantly outperformed batch fermentation, achieving a final OA titer of 260.1 ± 4.8 mM, a theoretical yield of 0.194 g/g and r_P_ of 0.061 g.L^-1^.h^-1^. A detailed comparison of the final product titers (mM), maximum attainable theoretical yields (Y_P/S_), and product formation rates (r_P_) for both operation modes is presented in Table 2.

## Discussion

This study systematically investigated biogenic OA production using submerged fermentation by filamentous fungi species, in particular *Aspergillus niger*, focusing on the interplay between strain selection, pH control, carbon source, and fermentation strategy. The findings of this study confirm that these factors are critically interconnected and must be co-optimized to maximize product yield. By integrating these variables, this work establishes a more comprehensive framework for OA fermentation than previously reported in disparate studies.

### Strain comparison test

Comparative study of strains confirmed that biogenic OA production is strain-dependent, as previous investigations also indicated considerable disparities in theoretical yields across various strains in organic acid fermentation (Ruijter et al. 1999). Similarly, over the course of 240 h, ATCC1015 secreted higher OA (71.0 ± 27.7 mM at 240 h) compared to CECT2807 (37.9 ± 7.1 mM at 240 h). These findings also align with the preliminary studies, including Mendes et al. (2022), who reported 155 mM from sucrose medium ( 100 g/L) using the strain ATCC1015 within 7 days, ≈ 6.6 times higher than an engineered mutant lacking *OafA* (oxalate titer ≈ 23.27 mM) (OA repression factor) (Mendes et al. 2022).

**Table 3:** Comparison of volumetric product formation (r_P_ with the preliminary findings concerning the oxalic acid generation across different scales and fermentation modes)

Operation	Scale	r_P_ (g.L^-1^.h^-1^)	Reference
Batch	STR (10 L)	0.036	This study
Fed-batch	STR (10 L)	0.061	This study
Batch	Shake flask	0.051	This study
Batch	STR (10 L)	0.283	Bohlmann et al. (1998)
Batch	Shake flask	0.104	Cameselle et al. (1998)
Fed-batch	STR (2 L)	0.26	Strasser et al. (1994)
Batch	STR (10 L)	0.375	Cameselle et al. (1998)
Batch	Shake flask	0.142	Santoro et al. (1999)

Nevertheless, large deviations across biological replicates (*n* = 3) were observed, particularly for ATCC1015, highlighting inherent biological variability of filamentous fungi fermentation (Bauer et al. 2022; Coban 2020). Variations in spore germination, pellet formation, hyphal branching, and oxygen diffusion gradients can be attributed to inconsistencies (Cairns et al. 2023; Veiter et al. 2018; Amenaghawon et al. 2024). Consequently, this morphological heterogeneity accounts for the drastic discrepancies in reported oxalic acid (OA) yields for ATCC1015, which range from 2 mM to over 150 mM in preliminary investigations (Brisson et al. 2016; Castro et al. 2023; Mendes et al. 2022).

Yet, confined to only three biological replicates, the present study struggles to completely isolate this inherent biological variability from extrinsic factors, such as sampling variance and experimental handling. Concerning the variations in pellet morphology, implementing advanced agitation or particle-enhanced strategies to stabilize pellet morphology, ensuring consistent leaching agent quality across industrial batches (Coban 2020; Mandal et al. 2005). Therefore, meticulous and systematic investigations comprising larger reproducibility studies are required to decouple these effects and standardize the biosynthesis of OA by ATCC1015.

### Influence of carbon source

The results suggested in Fig. 3 that the choice of a carbon source significantly influenced the OA secretion. Accordingly, glucose proved to be the most effective substrate, yielding 1.6-fold and 4.8-fold more OA than sucrose and lactose, respectively. This can be associated with the direct entry of simple sugars such as glucose into the glycolytic pathway, providing, hence, an efficient and forthright flux of carbon towards oxaloacetate, the immediate precursor of OA acid (Strasser et al. 1994; Walaszczyk et al. 2018). Used as a primary substrate, glucose can attain ≈ 75% of theoretical OA acid yield (Handayani and Suratman 2009). In contrast, more complex sugars such as sucrose and lactose require preceding enzymatic steps for break-down, thus diverting the metabolic flux in favor for the formation of other metabolites (Kubicek et al. 1988; Cameselle et al. 1998).

### pH influence and buffering dynamics

Our findings on pH regulation unequivocally demonstrated the decisive role of pH in the OA secretion. As shown in Fig. 2, in cultures where intermittent pH regulation was implemented to maintain the medium above pH 4, the final OA concentration attained 90.4 ± 5.8 mM. Conversely, the cultures without any pH control experienced a pH drop to approximately to 3, yielding a substantially lower concentration of 61.7 ± 1.6 mM. In support of the findings of Kubicek et al. (1988) regarding the oxaloacetate hydrolase *(OAH)*-dependent biosynthesis of oxalate at pH levels above 4, the findings presented in this study emphasize that maintaining the culture pH above 4 is not merely beneficial but essential for maximizing OA yields (Kubicek et al. 1988).

However, the buffering system employed during scale-up introduced a critical, albeit paradoxical, influence on fermentation kinetics. While 0.1 M phosphate buffering successfully mitigated acid-induced product inhibition, the surplus phosphate simultaneously favored exponential biomass growth at the direct cost of acidogenesis (Brown et al. 2018; Mandal et al. 2005; Manohar and Divakar 2002). This trade-off is starkly reflected in the fed-batch fermentation profile (Fig. 4a), where the onset of exponential OA secretion was delayed until the later stages of cultivation (≈ 312 h). This strongly suggests that substantial acid production only commenced once the initial phosphate excess had been depleted, inducing a metabolic shift consistent with phosphate-starvation-driven acid synthesis (Upton et al. 2017).

Furthermore, maintaining the bioreactor at a slightly acidic-to-neutral pH (≈ 6) to favor *OAH* activity inadvertently triggered the substantial co-production of gluconic acid (226.1±8.6 mM at 384 h). This pH range is highly conducive for *glucose oxidase (GOD)*, which rapidly oxidizes glucose into gluconic acid (Cameselle et al. 1998). While this did not hinder the absolute OA titer, it presents a downstream processing complication for selectively separating these two organic acids in industrial applications. Consequently, future investigations should focus on decoupling these effects by evaluating alternative buffering systems that facilitate high oxalate titers while minimizing biomass diversion. This should be complemented by systematic profiling of the phosphate-retardation effect to identify the precise phosphorus threshold required for a rapid metabolic switch from primary growth to secondary acidogenesis.

### Large-scale submerged oxalic acid fermentation

The scale-up comparison between batch and fed-batch operations provided critical insights into operational determinants in the large-scale submerged fermentation of OA. As the results in Fig. 4a suggested, the fed-batch strategy proved to be the most effective, achieving the highest final OA concentration (260.1 ± 4.8 mM) and maximum attainable theoretical yield $Y_{P/S}$ (0.195 g/g). By intermittently feeding of the substrate through pulsed feeding in 72 h of cycles, the residual glucose concentration was maintained below the inhibitory threshold of 20 g/L. This approach prevented carbon catabolite repression, mitigated severe pH fluctuations, and sustained a prolonged exponential production phase, a trend also observed in other organic acid fermentation processes (Schmitt et al. 2022; Demir et al. 2021).

Conversely, batch fermentation on a larger scale (10 L) exhibited a prolonged lag phase, with substantial oxalate secretion only commencing after ≈192 h, which can be attributed to inhibitory influences such as drastic pH fluctuations needed constant monitoring and control, and high initial glucose loading (150 g/L). What is more, high substrate loading can undermine oxygen transfer through osmotic pressure and increased viscosity, thus hindering organic acid generation (Xue et al. 2021; Anastassiadis and Rehm 2006). As indicated in Fig. 4a, the exponential production phase commenced only after the residual glucose concentration fell below ≈ 20 g/L.

When compared with the previous reports as outlined in Table 3, the volumetric product rates (r_P_) obtained from various scales and mode of operations in this study were substantially lower. This variance can be attributed to distinct disparities in experimental parameters, adopted feeding strategies, bioreactor geometries, and the inherent metabolic capacities of the specific fungal strains employed (Sitanggang et al. 2010; Keitel et al. 2024). However, an internal comparison between the small-scale batch and large-scale fed-batch cultivations demonstrates a highly successful bioprocess translation. By implementing strict pH control, strategic nutrient feeding, and an extended production phase, the scaled-up bioreactor system facilitated a substantially higher volumetric productivity for oxalate secretion compared to the preliminary flask data.

### Conclusions

This study underscores the decisive parameters governing biogenic OA generation via submerged fermentation. Among the evaluated strategies, fed-batch operation combined with intermittent pulsed feeding and robust pH control above 4.0 proved most effective, achieving a highly consistent titer of 260.1±4.8 mM with *A. niger* ATCC 1015. While the volumetric product formation rates were lower than highly specialized literature benchmarks, the primary goal of this research was not to maximize absolute yield at the cost of operational fragility. Rather, this work successfully established a simplified, robust framework suitable for direct integration into industrial critical metal recycling. By avoiding the extreme complexity and high capital expenditure of specialized fermentation systems, this optimized fed-batch method provides a reliable and sufficient supply of the biogenic agent necessary for critical metal (e.g., Gallium) leaching, offering superior industrial relevance for real-world e-waste recovery applications.

## Supplementary information

Supplementary material is available.

## Supplementary Information

Below is the link to the electronic supplementary material.

Supplementary file 1 (pdf 133 KB)

## Data Availability

All data generated or analyzed during this study are included in this published article and its supplementary information files.
