# Supplementary material for: Fermentation-based oxalic acid production aimed at sustainable critical metal recovery from electronic waste
Source: Appl Microbiol Biotechnol. 2026 Sep 23;110:284. doi: 10.1007/s00253-026-14040-4 (PMC13601489; doi:10.1007/s00253-026-14040-4)
Supplement: Supplementary file 1 [file 253_2026_14040_MOESM1_ESM.pdf]

Supplementary Information for:

Fermentation-Based Oxalic Acid Production for Sustainable Gallium Recovery from Electronic Waste

Aylin Nur Erkmen, Roland Ulber, Thomas Juestel, and Mirjam Altendorfner

S1. Supplementary Material

Instrument:Capel 105M S/N 1506
Capillary cassette and capillary
Number : 1166.3
Purpose : Organic acid
Internal diameter, µm: 50
Total length, cm: 50
Effective length, cm: 40
Coating : no
Temperature: 20.0 °C
Wave length: 254
Sample: Fed-Batch_384h_1000_#n
Dilution: 100.0
Buffer: Buffer for Analysis
Calc. method: Absolute calibration.

Program:
1.Rinse. From 3 to 1, t= 63 sec, U= 0 kV, P= 1000 mbar.
2.Injection. From 10 to 2, t= 5 sec, U= 0 kV, P= 30 mbar.
3.Analysis. From 4 to 2.
3.1. t= 419 sec, U= -25 kV, P= 0 mbar, W= 254 nm.

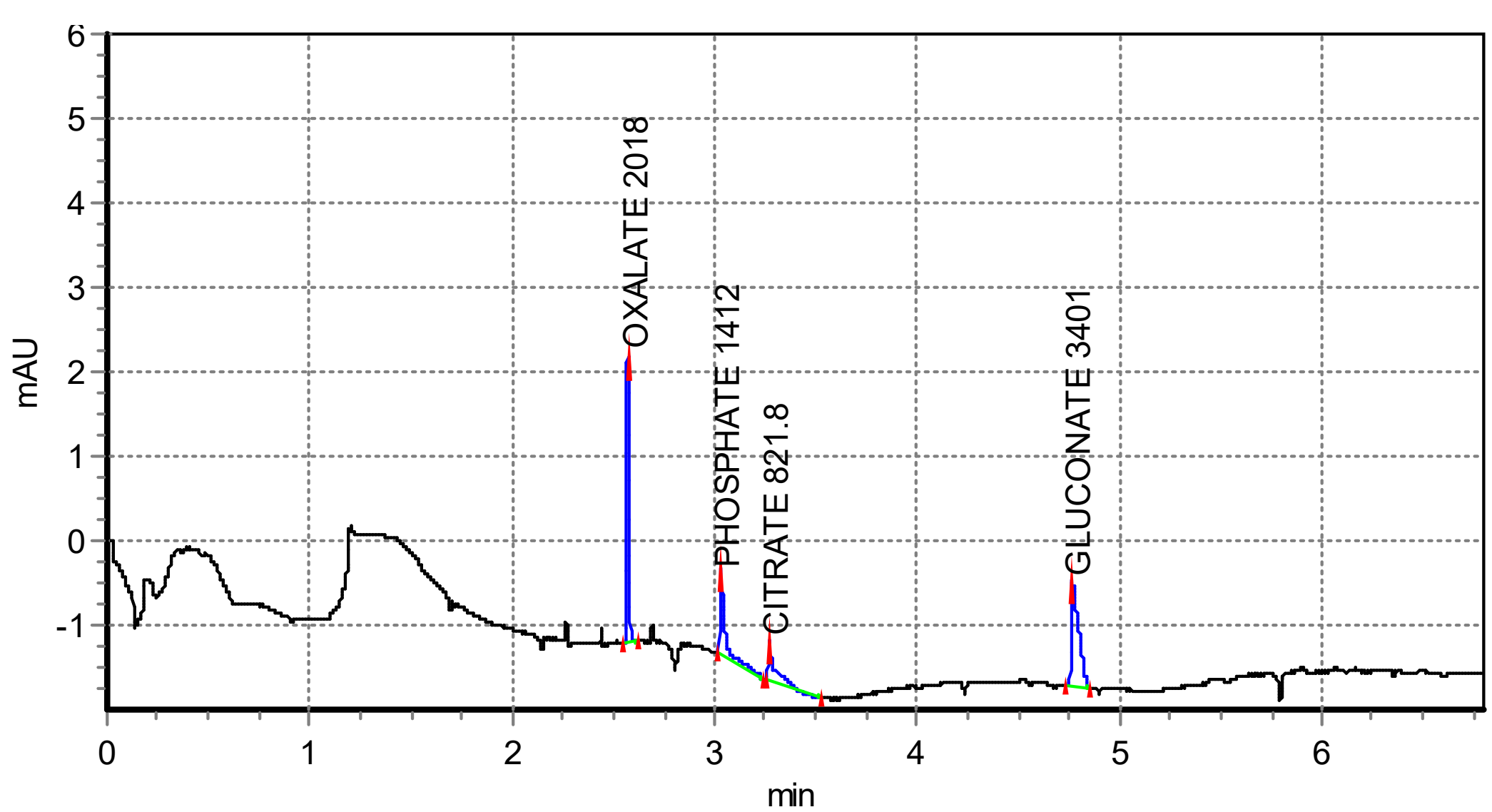

Figure S1. Electropherogram indicating the detected organic acids and anionic peaks in the supernatant of 384-h Fed-Batch fermentation (1:500 Dilution) by capillary electrophoresis. Capillary, 60 cm, capillary diameter 0.075 mm; temperature at 25°C, 5s of sample injection (30 mbar), -25 kV voltage, negative polarity, indirect detection at 254 nm using a buffer solution consisting of chromium (VI) oxide (10 mM), diethanolamine solution (30 mM), and Hexadecyltrimethylammonium hydroxide (CTA-OH) (3 mM)

N	Time	Component	Height	Begin	End	Area
1	2.572	OXALATE	3.395	2.550	2.625	21.82
2	3.028	PHOSPHATE	1.018	3.012	3.237	19.15
3	3.270	CITRATE	0.479	3.250	3.522	11.30
4	4.767	GLUCONATE	1.270	4.742	4.860	32.36
